# ‘Will I wear purple?’—a school arts-based research project in the UK to disseminate findings from a qualitative evidence synthesis about living to an extreme age

**DOI:** 10.1093/ageing/afad051

**Published:** 2023-06-26

**Authors:** Francine Toye, Alexa Cox, Cathy Jenkins, Karen Barker

**Affiliations:** Physiotherapy Research Unit, Nuffield Orthopaedic Centre, Oxford University Hospitals NHS Foundation Trust, Oxford, UK; Nuffield Department of Orthopaedics, Rheumatology and Musculoskeletal Sciences (NDORMS), University of Oxford, Oxford, UK; Art Department, Cheney School, Oxford, UK; Physiotherapy Research Unit, Nuffield Orthopaedic Centre, Oxford University Hospitals NHS Foundation Trust, Oxford, UK; Nuffield Department of Orthopaedics, Rheumatology and Musculoskeletal Sciences (NDORMS), University of Oxford, Oxford, UK

**Keywords:** qualitative research, arts-based research, ageing, school, community, older people

## Abstract

**Background:**

a change in attitude towards ageing is needed. Arts-based research (ABR) refers to the use of any creative art in research. ABR can provide an environment to reflect on challenging social issues and has the potential to make lasting impressions.

**Objective:**

we aimed to explore the use of ABR to disseminate findings from a qualitative evidence synthesis exploring what it means to live well beyond the age of 80.

**Design:**

ABR using art as a stimulus for recorded discussions and written annotations.

**Setting:**

a mixed catchment state secondary school in the UK.

**Subjects:**

fifty-four secondary school pupils aged 14–15. The majority identified as female (ratio 5:1).

**Methods:**

school pupils created artwork to represent themes about ageing drawn from a qualitative evidence synthesis. The artwork was a stimulus for recorded discussions. We used thematic analysis to develop themes about children’s response to ageing.

**Results:**

we developed six themes. Pupils found comfort in recognising that old age can be lived well; they began to see themselves in the older person; they explored the ambiguous nature of memory; they highlighted the dangers of disconnection; they affirmed a need to restore connection with elders and they recognised the need to cherish time and live meaningfully.

**Conclusions:**

this project encouraged pupils to think about what it means to grow old. ABR has the potential to contribute to a more positive relationship with older people and towards ageing. Research stakeholders should not undervalue the potential power of shifts in perspective for powering social change.

## Key Points

- Arts-based research (ABR) provides an environment for reflection on ageing issues and can make lasting impressions.
- ABR has the potential to contribute to a more positive relationship with older people and towards ageing.
- Through ABR, young artists began to see themselves reflected in older people.
- Through ABR, young artists recognised the dangers of disconnection and the need to nurture (re)connection with elders.
- Through ABR, young artists recognised the need to cherish time and to live their lives meaningfully.

## Background

Arts-based research (ABR) is the use of creative art in any phase of a research process, either for knowledge creation or dissemination [1, 2]. ABR can add value to research and is closely aligned with qualitative methodologies. First, it has a ‘democratising impulse’ [3]: it can include marginalised voices [4]; it is accessible [4–8]; it facilitates non-hierarchical relationships [5] and it invites participation and inclusion [9]. Second, ABR can provide a ‘safe’ space to explore challenging topics [6]. Third, ABR offers an ‘aesthetic’ way of knowing [4] which is ‘visceral, emotional and psychological’ before it is intellectual [6]. As such, it can make lasting impressions [3, 6] and become ‘part of one’s autobiography’ [10]. Fourth, ABR can move, perturb and unsettle [1, 6], facilitate compassion [4, 7] and encourage ‘humanly sensitive care’ [11]. This ‘transformative’ aspect is supported by a review of ABR, which highlighted insights for policy from ABR [5].

We aimed to used ABR to disseminate findings from a qualitative evidence synthesis exploring lived experience beyond the age of 80. This synthesis found that quality of older adult life hinged on being connected, yet also maintaining autonomy; that time was experienced as finite yet infinite; that it was important to live in the moment, yet also to ‘transcend’ the moment through reminiscence [12].

Preparing for an ageing population is integral to the United Nations 2030 Agenda for Sustainable Development, and some suggest that a shift in attitudes towards age is needed [13]. There are an estimated 1.8 billion young people aged 10–24 globally, and the World Health Organisation advocate them as a ‘powerhouse of human potential’ to drive sustainable development goals [14]. We wanted to disseminate our review findings about ageing to school pupils. We called the study, ‘Will I wear purple?’ inspired by Joseph’s poem, ‘When I am an old woman I shall wear purple’: ‘I shall . . . run my stick along the public railings and make up for the sobriety of my youth’ (Joseph, 1961): this fitted our intention to encourage pupils to think about what it was like to be an older person and to challenge age stereotypes of older age.

## Method

We contacted the head of creative arts at an urban state secondary school (1,480 pupils) who agreed to incorporate the project into their art curriculum. At that time, the school had a mixed socio-economic catchment: 33% first language was not English; 30% were eligible for free school meals; 17% had special educational needs support. Pupils and parents were sent information about the study. This was followed by a classroom presentation facilitated by the principal investigator (PI), where pupils could ask questions. We obtained parental consent via opt-out in accordance with approved ethical procedures for research in schools. We also collected assent from pupils, all of whom were aged 14–15.

The PI asked pupils to create and annotate a piece of artwork which represented one (or more) of the review findings. The PI attended six 2 hour art classes to collect observational and interview data as the pupils worked. As ageing may have been a sensitive topic for some pupils, the PI discussed potential issues with the classroom teachers prior to data collection and also asked if there were pupils who preferred not to discuss their art. The PI checked that each pupil was happy to talk, and to be recorded, and did not record without permission. Interviews were unstructured: the PI circulated as the pupils worked, asking about their art development and views about the topic. To facilitate anonymity, no names or sensitive information were recorded. Pupils worked on this project for one school term. Art boards and written annotations were photographed, and the data were transcribed and uploaded onto NVivo software for qualitative analysis.

The art was a stimulus for data production, and the data were the transcripts and annotations. The data were coded, and codes were organised thematically around a central idea through constant comparison [15]. Three researchers discussed the themes, codes and supporting data, agreed upon a thematic description and chose narrative exemplars. The PI curated artwork into an online exhibition (https://youtu.be/JjN_1m8fDDI) and presented this to the advisory team. For the purposes of publication, we selected a small number of pieces that exemplified the ideas. We gave pupils the opportunity to comment on the exhibition and to remove artwork.

## Results

Sixty-one pupils were invited and no parent withdrew consent. Seven pupils did not assent: 35 assented to use spoken words, art and annotations; 19 assented to use art and annotations; 3 requested that their art remain anonymous. The majority identified as female (ratio 5:1). We present each theme illustrated with narrative data. To protect the anonymity of pupils, we do not provide identifying information.

### I can see that you are vital, and this gives me comfort

The theme is underpinned by understanding that older people possess ‘vitality’. The pupils described vitality as the capacity to enjoy life: you can ‘still be old and happy’; ‘appreciate life’; ‘live to your full potential’; ‘have good times’; ‘make memories’; ‘have fun’. They described the bright side of getting older and the possibility of having ‘some of the best times of your life’.

These photos are taken to show the happy side of growing old . . . they show older people having a good time or going out and doing activities. This shows that being old isn’t all about staying inside and being lonely, it can be some of the best times of your life.

Vitality incorporated a sense of seeing ‘the child in your eyes’: ‘you still want to have fun’; ‘you can still act like a child’; ‘you can still be silly’; ‘you are still young at heart’. There was a sense that being ‘young’ was not about chronological age. Pupils felt that age did not define ‘you’: ‘you are still young on the inside’; ‘the child is within’; ‘your outside has changed but not the inside’.

It shows after all those years that have passed . . . and even though the outside has changed she still likes the same things . . . [the hat] is still special to her . . . it still stays vibrant colours because it is still staying alive . . . after all those years she is still a bit childish because she still likes the hat.

**Figure 1:**
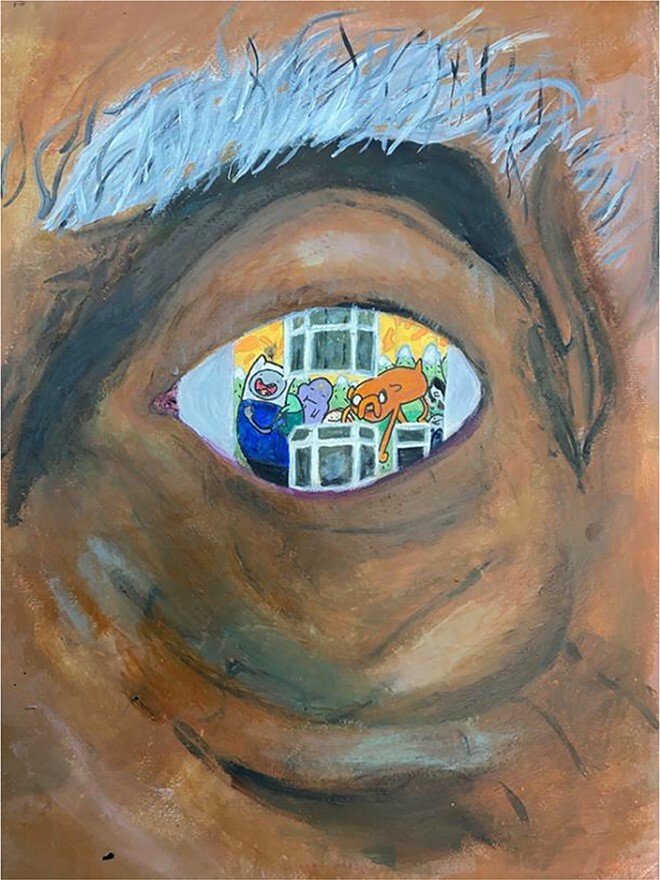
I can see the child in your eyes: ‘because the outside of you has changed it doesn’t mean you stop being yourself’.

**Figure 2:**
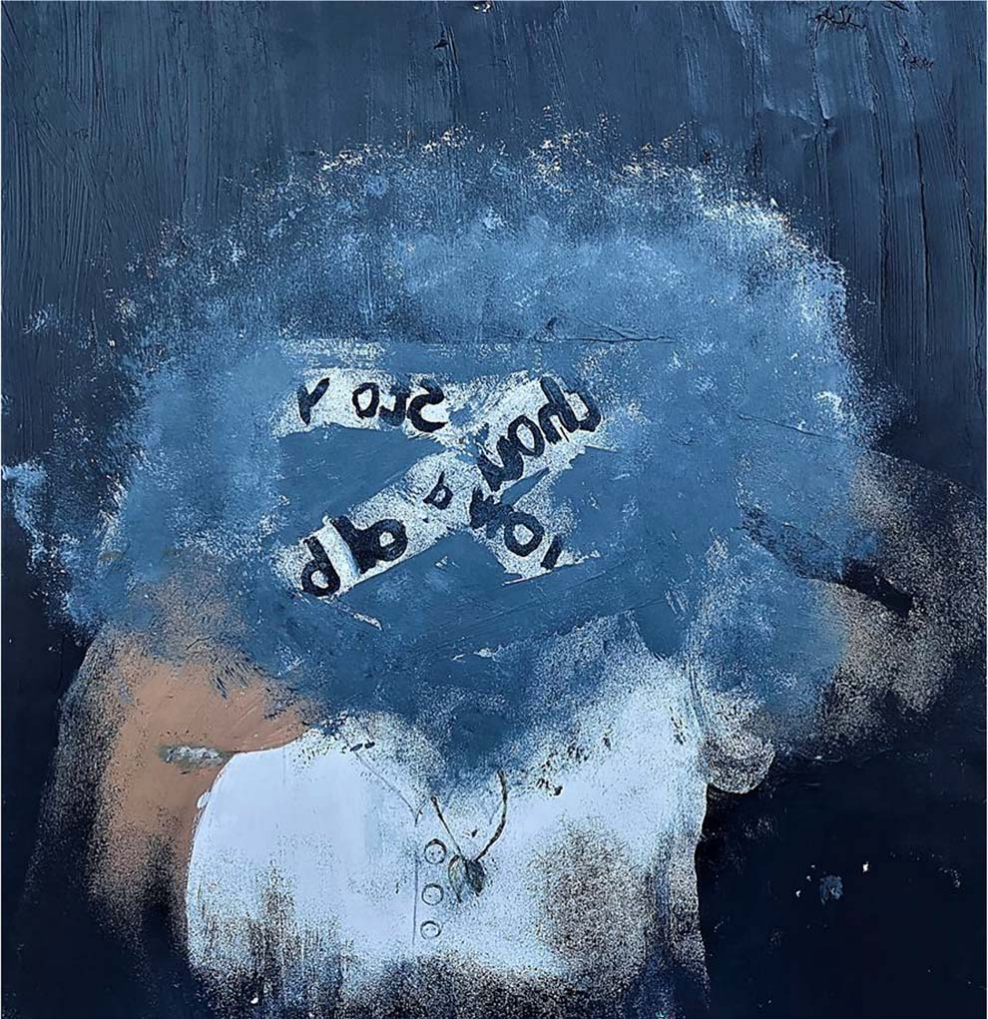
You have been hidden behind a mask: ‘you don’t see their talents, their memories and their identity’.

Vitality incorporated a sense of being ‘beautiful’ and of growing in beauty: ‘beauty is from the inside’; ‘all people are beautiful in their own way’; ‘I can see beauty in age’; ‘your eyes remain beautiful’; ‘beauty is in the eye of the beholder’. Some explored the value of ‘embracing imperfections’ and focusing on inner beauty.

This art piece inspired me to work more with older people and really start to embrace your imperfections instead of covering them up with make-up or clothes. As cheesy as it may sound, I do think that all people are beautiful in their own way, and we will embrace our appearance.

Vitality also implied personal growth and becoming ‘more complete’: ‘you have grown wise with age’; ‘you have lived through things’; ‘you have seen the world change’. The pupils described how older people had lived longer and therefore had more time to listen, observe and understand what was important: they ‘see things that we don’t see’; they ‘see things as they are’. They described learning from elders: ‘you can help us to grow’; ‘you are wise’.

We can learn a lot from mature people . . . we don’t appreciate them. If you listen to their stories and you listen to what they’ve gone through you can really learn something . . . they’ve lived a lot more than us so there is a lot more to learn.

Wisdom incorporated finding ‘peace’, ‘acceptance’, ‘freedom’ and even becoming ‘carefree’: ‘you no long longer have to worry about exams or getting a job’; ‘you have time to watch the world go by’; ‘you don’t have the time or energy left for chaos’; ‘worries of life have lost significance’, and you can now ‘focus on what you enjoy’. There was a sense of using time wisely before it ran out.

You find peace because you know you’re getting close to the end of your life . . . and most people have fulfilled what they want to fulfil and they can find peace and relax, and realise what went well with their life, like maybe cancel things that you don’t want in your life and just find peace.

Some told us that discovering that it was possible to be *vital* when old gave young people ‘comfort’ to face the inevitability of ageing: ‘It was a bit scary to think of getting old’; ‘it doesn’t seem so scary now’; ‘It makes me feel brave’.

At the beginning I was quite scared of ageing . . . this makes me feel less negative about it . . . because you can be old and not be consumed by the fears of how close you are to dying . . . You can still have fun and be childish.

### I see a person like me beneath their ageing exterior

This is underpinned by a growing awareness that beneath the exterior, the older person is ‘just like me’, ‘hidden behind a mask of age’. Pupils put themselves in the older person’s shoes and began to see life through older eyes.

Pupils recognised that an older person’s experience and talents often remain hidden by stereotypes: ‘we have noticed the signs of age and not the person’; ‘you are labelled as old’; ‘we don’t see your talents, capabilities or knowledge’; ‘we don’t see the lives that you have lived’; ‘we do not hear what you have to teach us’.

I am focusing on . . . hidden treasures, I think old people are often stereotyped when you first see them and you don’t see their talents, their memories, and their identity and I wanted to look at that . . . first look you just see old person . . . [but you are] a handyman, a reader, a carer, a scientist, a rugby player, a pianist, a father.

Some recognised that older people might have led, and be leading, challenging lives, and that these challenges might resonate with younger people. For example, ‘older people can also suffer with poor mental health’; ‘older people can also live hard lives, depression, loss, and loneliness’; ‘it is not just a problem for young people’.

I have decided to focus on the more mental side of ageing for example depression from loneliness or someone important dying. Mental health is usually only thought about in young people and I’m going to focus on things not really mentioned.

Pupils described how they had started to see the world through an older person’s eyes: ‘I am now seeing the person: what they once were and how times have changed’. Some had become interested in past lives and had been surprised about what they had found out from grandparents: ‘I want to find out about their lives’; ‘it is good to put yourself in someone else’s shoes’. Pupils told us that they realised ‘I am that person’; ‘I will be old one day’; ‘you were once like me’.

I tried to put myself in the mind-set of an ageing human, to think of growing old from their perspective. I thought about what such a person would find important and thought of their memories . . . their experiences . . . I think just understanding that they had a childhood just like us. That they were our age once.

### Memory is ambiguous, it can be a blessing and a curse

This theme explores the ambiguous and constructed nature of memory. Pupils described memory as serving a changing function with age. Some felt that memory was reflective: ‘you reflect on what life was like’; ‘you think about how things have changed’; ‘a river of memories connects your life’; ‘you reflect on time gone by’; ‘you look back on your journey’.

Some people might look out of the window and think of life and what they want to do . . . Older people might be thinking of memories and what they used to do, and what they can do now . . . they can see the difference of what things were like then and now.

Good memories might ‘offer protection through hard times’ and ‘make you smile and give strength’. Pupils described how older people ‘gather treasured memories’, such as photographs or letters, that ‘tell a story of your past’, ‘bring back fond memories’ or ‘hold and trigger memories’. These treasured objects were tangible physical memorabilia which can ‘connect us with someone’ distant or lost. Pupils described their own connection with family members through shared memories: ‘we are still creating memories together’; ‘your memories will become my memories’.

Certain things or memories can protect us like umbrellas protect us from rain . . . umbrellas protect you from bad weather, don’t they? . . . kind of like protection.

Pupils recognised the deep loss of losing memory: ‘identity drifts away with your memories’; ‘memory starts to tear apart’; ‘you can forget who you are, and who other people are’. Some told us how dementia can ‘confuse and bewilder’ as memories were lost ‘piece by piece’.

It’s almost like distortion of memory like as you get older you kind of forget . . . as you get older your memory start to . . . tear apart . . . You start to forget things . . . chaos of the mind, disruption . . . memory loss.

**Figure 3:**
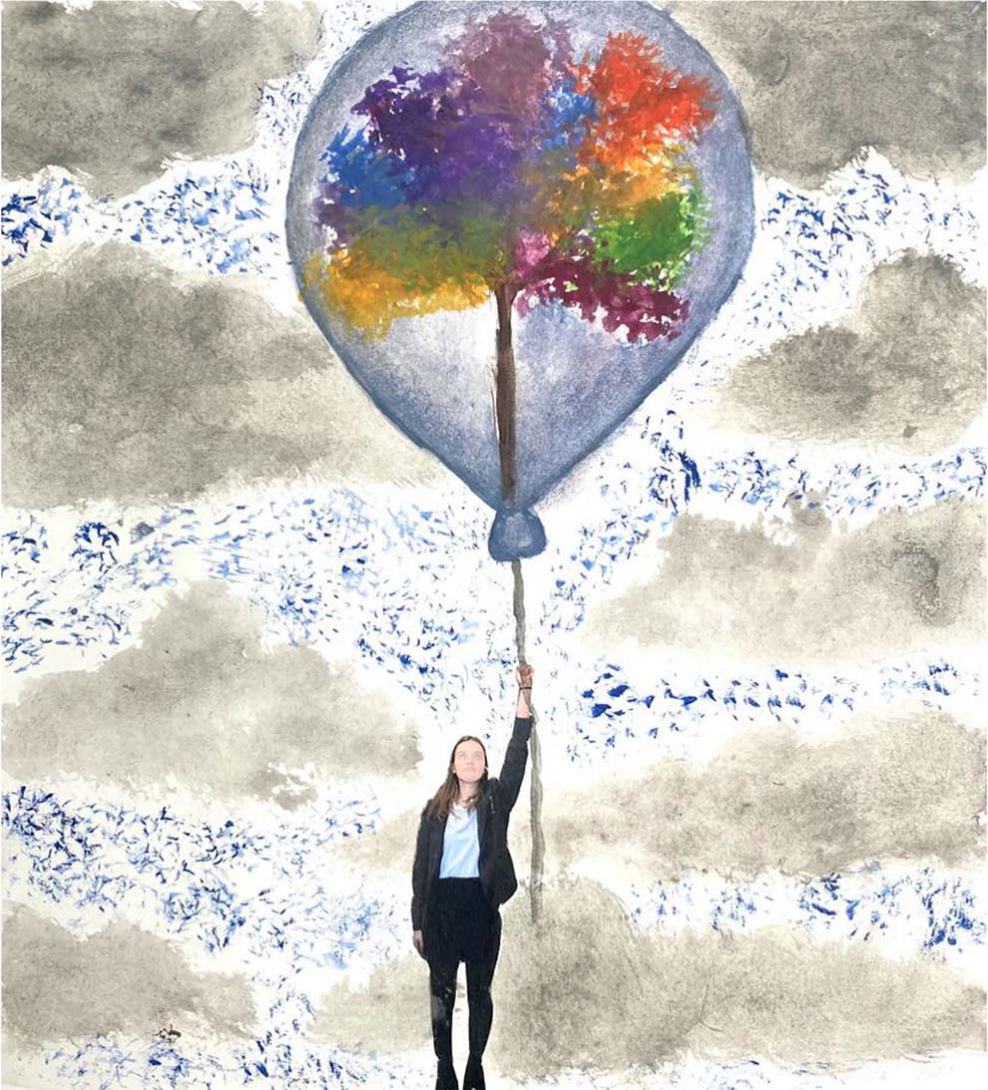
You gather treasured memories: ‘memories can protect us like umbrellas protect us from rain’.

However, memories could be a double-edged sword: ‘memories can be good and bad’. Pupils felt that there were things that you might not want to dwell on, as ‘life is not all roses’: ‘the road can be hard for some’; ‘some have lived through wars and hardship’; ‘It’s not all light and sunshine’. They recognised that there might be things that might ‘drag you down and make you sad to think about how things used to be’.

**Figure 4:**
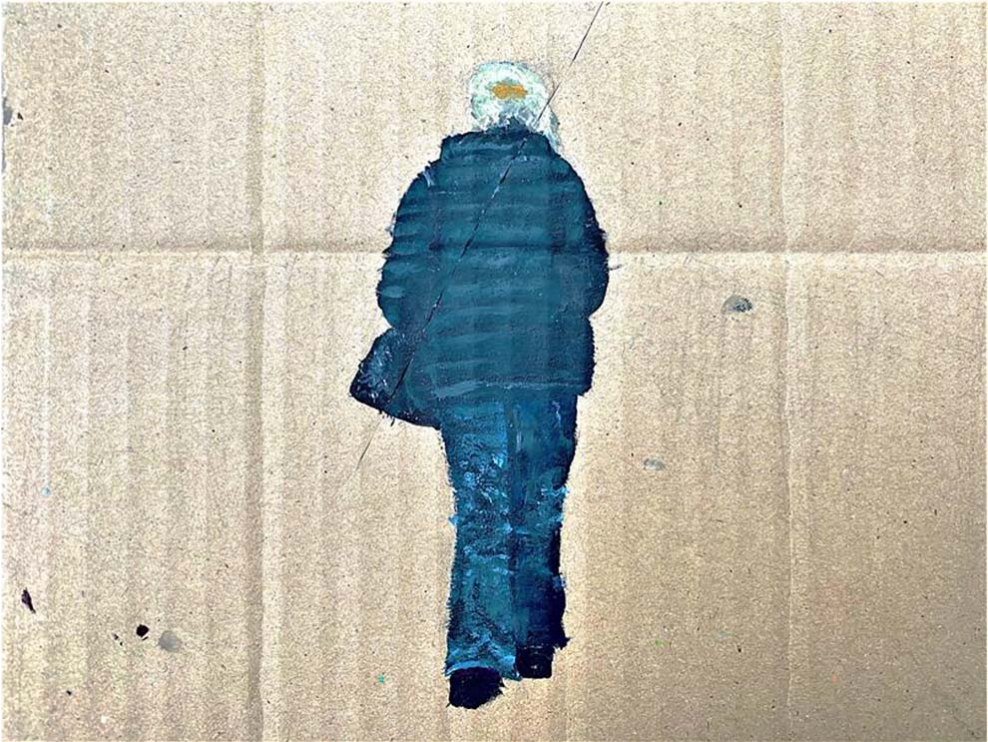
You have been overlooked: ‘most people don’t catch what old people are saying’.

It kind of depends on how you interpret it, cos for me I think of [memories] kind of dragging you down . . . I think when you’re older thinking back to when you are young when you can do all these things . . . I think it might be quite sad looking back on those memories thinking that you can’t do it anymore.

Pupils described how memory is selective, subjective and contextual, rather than ‘real’: as such, memory could ‘deceive and distort’. They described how two people might remember the same event differently. Memory was framed as fluid and changing: some memories might ‘fade’, where others might be ‘locked in and stored forever’.

Even the same event, two people would have very different memories of how it looked . . . two different places, and two different skies . . . you could probably say that one person would see the overcast day and the other person would see the sunset.

### Older people are becoming disconnected

This theme explores social disconnection: ‘you are slowly fading and disappearing from the world’; ‘you are being erased’; ‘you are becoming unrecognisable’.

The face of that person is slowly disappearing bit by bit . . . [it] is sad because it is showing a person almost disappearing from the world. . . faded and disappearing . . . it makes me feel as though someone is almost rubbing out the portrait.

Disconnection was linked to a failing body and its diminishing effect on geographical and social space: ‘eyesight fails you’; ‘you can’t do as much’; ‘you can’t go out’. Pupils described a diminishing enjoyment whereby life could become dull, monotonous and mundane.

When you’re older you can’t do as much as everyone else. You are limited to do things because of your physical body. Loss of eyesight could play a part in mental ageing or fading away from society.

Pupils felt that older people were overlooked and devalued: ‘you are no longer part of community’; ‘you don’t feel valued’; ‘you are not heard’; ‘you are left out’; ‘you are an outsider; ‘you are losing touch with the world’. Some were even concerned that older people might be ‘mistreated’ by others.

Old people are still part of the community and yet not part of the community . . . like dead leaves on the tree . . . they’re still on the tree surrounded by the other green leaves. They are still people, and they are paying taxes and things like that, and buying shopping and stuff . . . but they feel they are not doing anything to help the community because they are retired . . . and not valued.

Pupils recognised that disconnection was exacerbated by losses and of feeling ‘left behind by loved ones that have passed’; ‘you feel emptiness and loneliness’; ‘you feel trapped and alone’.

The cages show how some old people . . . feel like they’ve been separated or left behind by the rest of their generation that have passed away. Feeling diminished can be feeling trapped in your own life and even though you’re alive you feel more dead than the ones you have passed away.

### We are all connected and should take care of each other

The theme described connection as integral to a vital life, and some felt an obligation to nurture connection with older people: ‘*we* are all connected to each other’. Pupils drew on their own family experience to describe the importance of connection; ‘My family is vital to me and I am vital to them’; ‘we don’t appreciate what we are part of’. They described powerful and meaningful connections to family and to humankind: ‘we are part of a whole; ‘we stand together’. Some recognised that through connection came solidarity and growth: ‘we grow and blossom through connections’; ‘our lives intertwine and have an impact on each other’. They also recognised the deep roots that accompanied human connection: ‘we go in and out of connection over time, but connections remain’; ‘we stand together’.

I have painted acrylic flowers where the man’s footsteps would have been . . . it shows that the way in which the man’s life has impacted on others *matters* . . . where the man has walked beautiful flowers have bloomed . . . through relationships we bloom and blossom, connection is beautiful like the flowers.

Some described the impact of the art project on reconnecting with elders; ‘[it] has opened a new conversation’; ‘I should spend more time with old people’; ‘I have started to notice older people out there’; ‘it’s not fair to leave them alone’; ‘be friendly’; ‘respect them’; ‘make them part of everyday life’; ‘understand what they are going through’; ‘help out’; ‘smile’; ‘brighten up their day’.

I feel like everyone should be important and everyone is important. . . It is really odd to see how someone can change [as they get old], so they are not really wanted, and not valued . . . Whereas I kind of feel that maybe we should do more to make people part of the everyday life.

**Figure 5:**
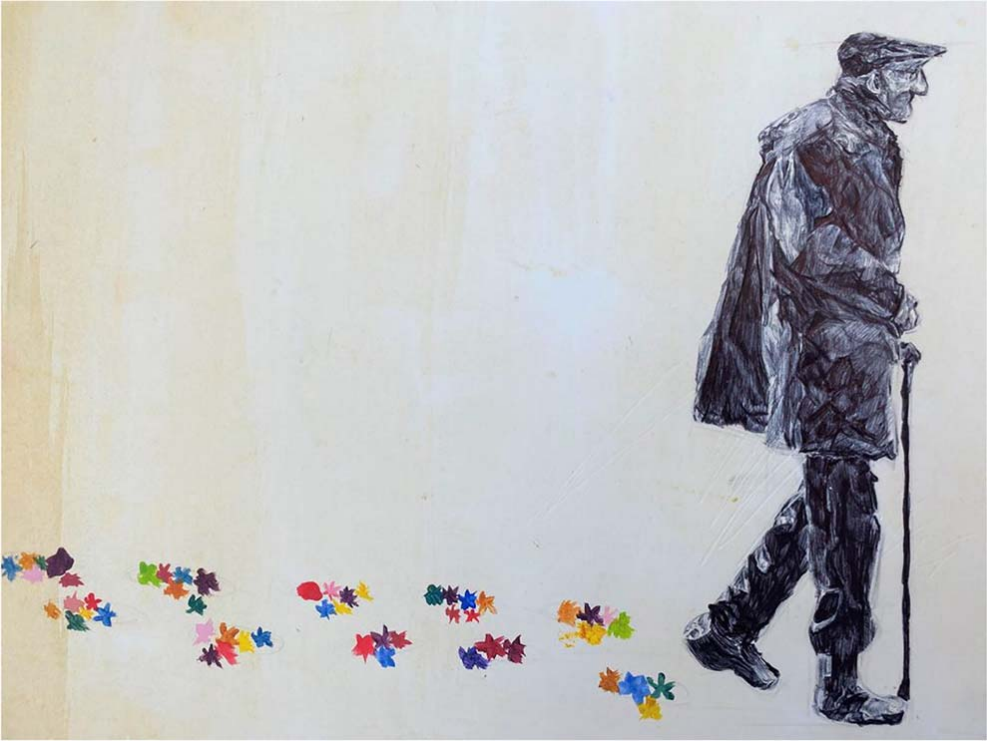
Connection is the source of vitality: ‘connection is beautiful like the flowers’.

### We are on a one-way journey and should make the most of it

The final theme is underpinned by human solidarity on the life journey. Pupils felt that old age was not a destination that you arrived at: ‘you don’t arrive at old age; it is just where you are’; ‘old age is just another stage in life’. They recognised the inevitability of change and ‘it would not be good to stay the same’: As such, getting old was just ‘the beginning of a new chapter’. There was also a sense that you might not anticipate or recognise the arrival of old age: ‘It doesn’t seem old when you get there’.

**Figure 6:**
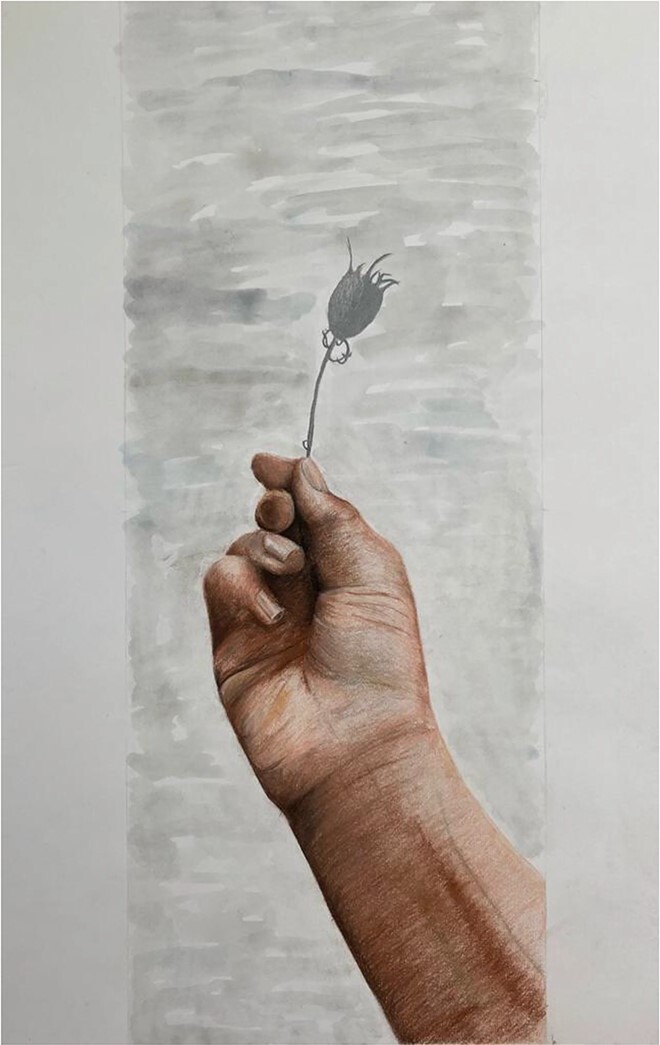
We are part of the fabric of nature: ‘*with nature, I can die, and just start growing all over again in a cycle*’.

As you get older you think ‘this isn’t such a big age’ because you are that age now. When you are little, you think that being 13 is really, really old, and then you get to 13 and you think well it’s not . . . So when you become older you don’t think, *old*, you think this is how old I am so it’s not a big deal . . . It is just how old you are.

Pupils described human beings as part of nature and as such subject to the natural laws: ‘we are all part of nature and the world around us’; ‘in nature, things die and replenish’; ‘journeys end and begin again’; ‘decay is part of the natural order’; ‘it is part of the cycle of life and death’; ‘things return to dust’; ‘the world is renewed’.

Everything is alive and then dies at least once, but with nature, I can die, and just start growing all over again in a cycle. Which only happens to plants, not to humans and animals, they are alive, die and then that’s it. No starting over in life.

Pupils represented skin and eyes to illustrate the process of ageing, and physical changes were framed as a sign that you have lived: ‘skin is a testament to the passage of time’; ‘you are weathered by time’; ‘[time] leaves its mark on your face and hands’; ‘your skin is a mark of how many years that you have lived’; ‘the things that you have done in your life show themselves in your skin’.

Old hands show the elderly getting fragile . . . and young hands together to show the contrast in the relationship between the elderly and young people in a family. The old hands look tired and show how many years the person has lived.

There was an acceptance that ‘all life comes to an end’; ‘this is part of living’; ‘you are living on the brink of life and death; ‘you are nearer to death than to life’; ‘death creeps up on you’.

[I have shown a] skeleton representing death because when you die your flesh will melt away, because ageing is kind of like getting closer to death, and it kind of shows being on the edge of life and death.

Some framed the human journey as ‘insignificant in space and time’: ‘time passes’; ‘seasons change’; ‘day becomes night . . . night becomes day’; ‘human life is one moment in the ebb and flow of time’; ‘we are insignificant in the passage of time’.

I was thinking the shell used to, there used to be something living inside it in the ocean . . . And I did some shells . . . I was just thinking that they used to be something alive in it and also the shells they all just turn into rock so it’s like a cycle.

Recognising the finite nature of the human condition, pupils acknowledged the need to make the most of the journey: ‘I have learnt that we should make the most of life’; ‘live life to the full while you can’; ‘make the most of the moment’; ‘cherish your time’; ‘be meaningful in what we do’; ‘stop the ceaseless worry’; ‘don’t put things off’; ‘you can’t turn back the clock’; ‘nothing lasts forever’. There was a sense that you can ‘never know what is around the corner’.

The match represents our life, once it starts burning, you cannot make the burnt part to its original state . . . if you focus on the burnt part, you lose the rest of the match . . . you can make the most of it and make endless energy. . . [time] goes in one direction. You cannot make the watch turn anticlockwise . . . you will lose your time trying.

## Discussion

We explored the use of ABR to disseminate findings from a qualitative evidence synthesis [12]. Some felt that project and been an ‘eye-opener’ which encouraged them to ‘think deeply about growing old’.

I feel like this whole like new idea . . . like [old age] is not really the end, it is more or less like the beginning of something, the beginning of a new chapter . . . You are not younger anymore and you started a new chapter in your life.

Living in an age-segregated society can lead to negative or unrealistic assumptions about ageing [16]. Although ageing and dying are inevitable, these ‘taboo’ subjects are rarely discussed [16, 17]. ABR can provide a forum to explore challenging topics. Our findings resonate with community projects using ABR to create space for younger people to explore ageing and dying. The Schools Project at St. Christopher’s Hospice, London, UK [17], brought together hospice patients with local pupils to promote healthy attitudes towards, death, dying, loss and transitions [17]. The project ‘normalised’ death as part of the life cycle and had a positive impact on attitudes towards age and dying. Similarly, the studio DöBra project in Sweden [16, 18], which used art to stimulate conversations about dying, death and loss between primary school children and elders, challenged negative age-related stereotypes [18].

Our project took place in one state secondary school with a mixed catchment, and other schools might bring different perspectives. We only included a school year (aged 14–15) as this fitted with the curriculum. Future studies might usefully include different school years. Our aim was to explore the use of ABR to disseminate findings in a classroom setting, not to create an intergenerational learning space. Future studies bringing together older and younger pupils might contribute to learning.

Our study highlights some methodological considerations. First, ABR faces the criticisms of interpretive methodologies. Scientific research is steeped in a tradition that is sceptical about creativity and interpretation, fearing that it introduces ‘bias’, threatens objectivity and obscures ‘truth’ [5, 9]. In contrast, interpretive qualitative research require us to recognise, embrace and challenge our situated position [15], and value unique perspectives for their role in sensitising us to ideas [19].

Second, ABR challenges our ideas about what evidence *is* [7] and highlights the ‘slippery distinction’ between knowledge creation and dissemination [6]. ABR highlights that knowledge is not just a product to be disseminated [4]. On the contrary, *knowing* is a dialectic process (not a product) that occurs at the interface of ideas. Although we used ABR to disseminate research findings, we found that the process of dissemination co-created an additional layer of knowledge.

Third, our project highlights the challenge of determining what ABR data is [5]. Although we used art as a stimulus for data (interviews and annotations) production, it was difficult to extricate the ‘data’ from the art process. The distinction between art *in* research (where art is a stimulus) and art *as* research (where art is the data) is not always clear-cut [1].

Fourth, there are currently no criteria for evaluating ABR or determining its impact [20, 21]. Impact might simply mean a ‘subtle shift in viewers’ perspectives’ [3]. For example, does it generate emotions: does it help the audience notice and understand the issues: does it generate internal dialogue and increase engagement: does it ‘move the audience to change in salient ways?’ [20].

Our study highlights ethical challenges [7, 10]. In ABR, there is a tension between the need to protect privacy and the need to recognise ownership of art: our pupils were simultaneously ‘artists’ and ‘participants’. Although we had assent from most pupils to name their work, some did not assent, and we made the decision with the classroom teacher to protect privacy, as an underlying principle of research. We contacted our university research ethics manager, who agreed with this decision. However, some might feel strongly that the pupils should be named, and argue that we have usurped their creations. This issue is unique to ABR, and future projects (and ethical evaluations) should consider, and be explicit about, the decision to name artwork. Another ethical issue linked to the ‘aesthetic’ way of knowing in ABR is the unique potential to unlock emotions [5]. Our study took place within the classroom under supervision of a teacher who knew the pupils. We also knew that other studies had explored end of life or death and dying with younger participants [16–18]. Although no incidents arose in the classroom, it is important to consider emotional repercussions beyond the classroom. While ABR can ‘break through, uncover, penetrate and reveal’, it is also ‘supporting, containing, sustaining and nourishing’ [22].

ABR ‘requires a willingness to be transformed and educated’ and encourages researchers, participants and audiences to look at things in new ways. Art requires interpretation: it is made, not found. It is perhaps the ambiguity of art that encourages the incubation of ideas, and it can therefore be a useful *heuristic device*, by allowing people to ‘learn by making discoveries for themselves, rather than being directed’ (Oxford English Dictionary). A poignant example of the transformative power of ambiguity in art is exemplified by an advisory members’ response to an illustration portraying an empty shell—‘there used to be something living in there’ (Supplementary Appendix 1): while the artist told us that they were representing the cycle of life, one advisory member was reminded of ‘a person wrapped in a shroud’. Our study demonstrated a powerful interplay between picture and words that transformed and left lasting impressions. The brain makes sense of art in ways that may support long-lasting impacts: this way of knowing may cultivate empathetic understanding, awareness of issues and ‘critical consciousness’ [2]. As such, ABR is closely aligned to interpretive methodologies.

Our findings indicate that ABR encouraged pupils to think about what it means to grow old, and we suggest that this has the potential to contribute to a more positive relationship with elders and towards ageing. Pupils found comfort in recognising that old age can be lived well; they began to see themselves reflected in the older person; they explored the constructed and ambiguous nature of memory and its centrality to a sense of self; they highlighted the dangers of disconnection of old age; they affirmed a need to restore and nurture connection with older people; they recognised the need to ‘cherish time and be more meaningful in what we do’. ABR can provide an environment to reflect on challenging social issues and has the potential to make lasting impressions. Research stakeholders should not undervalue the potential power of ‘shifts in perspective’ for powering social change.

## Supplementary Material

aa-22-1736-File008_afad051

## Data Availability

The datasets generated and/or analysed during the current study are not publicly available to ensure anonymity of participants. Examples of the artwork generated are available in an online exhibition (https://youtu.be/JjN_1m8fDDI).
