# Supplementary material for: ‘Will I wear purple?’—a school arts-based research project in the UK to disseminate findings from a qualitative evidence synthesis about living to an extreme age
Source: Age Ageing. 2023 Jun 26;52(6):afad051. doi: 10.1093/ageing/afad051 (PMC10294293; doi:10.1093/ageing/afad051)

**‘Will I wear purple?’ – A school arts-based research project in the UK to disseminate findings from a qualitative evidence synthesis about living to an extreme age**

Appendix 1: ‘there used to be something living in there’


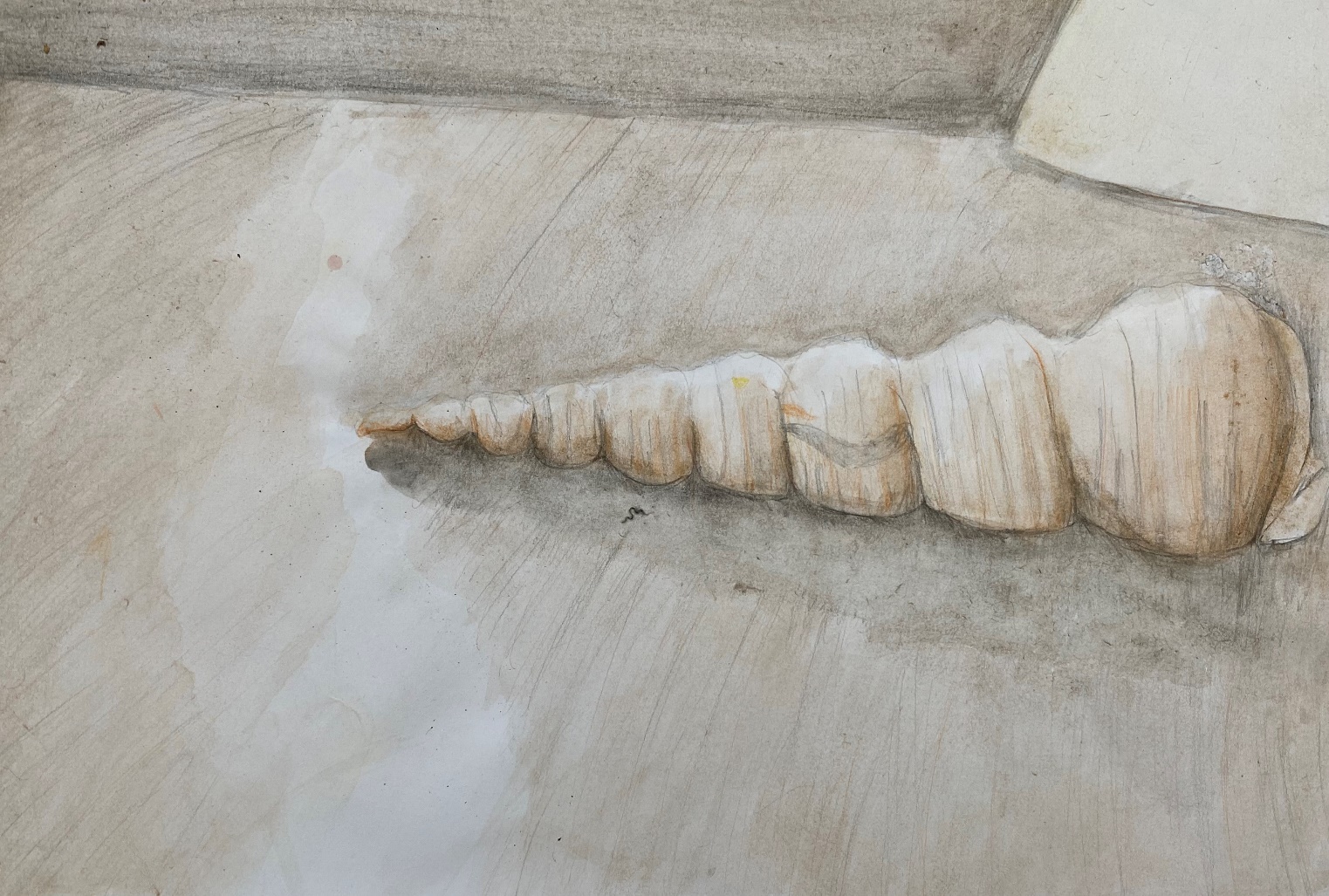

Supplement: aa-22-1736-File008_afad051 [file aa-22-1736-file008_afad051.docx]
